# DNA Methylation Profiles of *Tph1A* and *BDNF* in Gut and Brain of *L. Rhamnosus*-Treated *Zebrafish*

**DOI:** 10.3390/biom11020142

**Published:** 2021-01-22

**Authors:** Mariella Cuomo, Luca Borrelli, Rosa Della Monica, Lorena Coretti, Giulia De Riso, Luna D’Angelo Lancellotti di Durazzo, Alessandro Fioretti, Francesca Lembo, Timothy G. Dinan, John F. Cryan, Sergio Cocozza, Lorenzo Chiariotti

**Affiliations:** 1 Department of Molecular Medicine and Medical Biotechnology, University of Naples “Federico II”, Via S. Pansini 5, 80131 Naples, Italy; mariella.cuomo@unina.it (M.C.); giulia.deriso@unina.it (G.D.R.); lunalancellotti92@gmail.com (L.D.L.d.D.); cocozza@unina.it (S.C.); 2 CEINGE Biotecnologie Avanzate, via Gaetano Salvatore 482, 80145 Naples, Italy; dellamonica@ceinge.unina.it; 3 Department of Veterinary Medicine and Animal Productions, University of Naples “Federico II”, Via Delpino 1, 80137 Naples, Italy; luca.borrelli@unina.it (L.B.); fioretti@unina.it (A.F.); 4 Task Force on Microbiota Studies University of Naples “Federico II” of Naples, 80131 Naples, Italy; lorena.coretti@unina.it (L.C.); frlembo@unina.it (F.L.); 5 Department of Pharmacy, University “Federico II” of Naples, via Domenico Montesano, 80131 Naples, Italy; 6 Department of Psychiatry and Neurobehavioural Science, APC Microbiome Institute, University College Cork, T12 YT20 Cork, Ireland; t.dinan@ucc.ie (T.G.D.); j.cryan@ucc.ie (J.F.C.)

**Keywords:** DNA methylation, microbiota–gut–brain axis, *Zebrafish*, cell-to-cell heterogeneity, methylation profiles, epialleles

## Abstract

The bidirectional microbiota–gut–brain axis has raised increasing interest over the past years in the context of health and disease, but there is a lack of information on molecular mechanisms underlying this connection. We hypothesized that change in microbiota composition may affect brain epigenetics leading to long-lasting effects on specific brain gene regulation. To test this hypothesis, we used *Zebrafish* (Danio Rerio) as a model system. As previously shown, treatment with high doses of probiotics can modulate behavior in *Zebrafish*, causing significant changes in the expression of some brain-relevant genes, such as *BDNF* and *Tph1A*. Using an ultra-deep targeted analysis, we investigated the methylation state of the *BDNF* and *Tph1A* promoter region in the brain and gut of probiotic-treated and untreated *Zebrafishes.* Thanks to the high resolution power of our analysis, we evaluated cell-to-cell methylation differences. At this resolution level, we found slight DNA methylation changes in probiotic-treated samples, likely related to a subgroup of brain and gut cells, and that specific DNA methylation signatures significantly correlated with specific behavioral scores.

## 1. Introduction

Several lines of evidence have demonstrated the existence of complex and bidirectional interplay between the brain and the gut (gut-brain axis) [1,2,3]. The gut microbiota could be a significant environmental factor influencing brain function and, most importantly, the brain epigenome [4]. In turn, the epigenetic modulation of brain cells could represent the genomic integration of signaling originating from the gut microbiome [5]. In fact, the establishment of an equilibrated gut microbiota, especially during prenatal, early postnatal and infant phases, appears to be crucial to enabling correct brain development and mental health later in life [6,7]. An imbalance in the composition of the gut microbiota has been associated with numerous diseases, including neurodevelopmental, psychiatric and neurodegenerative disorders [8]. Based on these observations, microbiota-modulating strategies have been suggested as a potential novel therapeutic approach in the treatment of disorders related to the central nervous system (CNS), such as autism spectrum disorder (ASD) [9,10,11]. Several efforts have been conducted to disentangle the main routes connecting brain epigenetics and gut microbiota. This cross-talk is thought to occur both in direct and indirect manners. Some products of bacterial metabolism, such as butyrate and propionate, are well known key modulators of several epigenetic “erasers”, such as histone deacetylases (HDACs) [12,13]. Furthermore, DNA methyltransferases (DNMTs), the enzymes catalyzing the transfer of a methyl group on a CpG dimer, are highly sensitive to the availability of S-adenosylmethionine (SAM). SAM levels may be, in turn, regulated by the abundance of some molecules, including folate, which supports one carbon metabolism, provided also by gut microbial communities [14,15,16,17]. A pilot study [18] of DNA methylomes conducted on the blood of pregnant women revealed an association between bacterial predominance and epigenetic profiles: gut microbiota enriched by Firmicutes or Bacteroidetes correlated with the differential methylation status of gene promoters functionally associated with cardiovascular diseases, lipid metabolism, obesity and inflammation. The majority of the available data was obtained by correlative studies performed on rodents. Some studies describe the *Zebrafish* animal model as a promising model to study diseases linked to the functioning of the microbiota–gut–brain axis [19,20,21]. These fish possess a rich gut microbiota (GM) comparable to that of the mammalian [22]. In addition, the presence of major neurotransmitters and their receptors makes *Zebrafish* a suitable model to study the gut–brain axis [23]. We and others have recently demonstrated that the administration of *Lactobacillus rhamnosus* for 28 days in the *Zebrafish* model shoaled the behavior of the fishes and increased the expression levels of genes, including *Tph1A*, which is involved in serotonin synthesis, and the neurotrophic factor gene *BDNF* [19]. Both *Tph1A* and *BDNF* are thought to have a direct impact on behavioral control in Zebrafish [23,24,25]

In the present work, we analyzed the *Zebrafishes* gut and brain samples characterized in the previous study [19] in order to investigate whether probiotic-induced behavioral changes are associated with DNA methylation changes at the relevant loci. In addition to analyzing average methylation, we performed a high-coverage single-molecule DNA methylation analysis of the *Tph1A* promoter region using the bioinformatic pipeline ampliMethProfiler [26]. Using this approach, we detected tissue-specific differences and slight probiotic-induced methylation changes at *BDNF* and *Tph1A* promoters. Importantly, we found that behavioral variations induced by probiotic administration significantly correlated with DNA methylation changes at the *Tph1A* gene, both in the brain and in the gut.

## 2. Materials and Methods

### 2.1. Ethics Statement, Euthanasia and Animals

Four- to six-month-old male and female *Zebrafish* (*Danio rerio*) of the heterozygous “wild type” strain, obtained from commercial distributors (Carmar, Napoli, Naples, Italy), were used. All fishes were acclimated to the laboratory environment for at least 14 days in a 30 L tank in recirculating systems with deionized water. Fishes were fed twice daily with sterilized commercial food (Sera Vipagran, Heiensberg, Germany). The room, water temperatures and illumination were maintained according to the standards of *Zebrafish* care [19,27]. As reported by our previous studies [19,27], all fish were treated in accordance with the Directive of the European Parliament and of the Council on the Protection of Animals Used for Scientific Purposes (Directive 2010/63/EU), and in agreement with the Bioethical Committee of the University Federico II of Naples (authorization protocol number 47339-2013). Following behavioral testing, the animals were euthanized by immersion in overdose 500 mg/L^−1^ of 3-aminobenzoic acid ethyl ester (MS-222) buffered with sodium bicarbonate (1:2 ratio solution), to pH 7.4 (Sigma–Aldrich, St Louis, MO, USA), and brain and gut tissues were taken and stored at −80 °C.

### 2.2. Probiotic Administration 

Two experimental groups were evaluated: a control group (CTRL) and a probiotic-treated group (PROBIO). The control group (CTRL) was fed twice per day with the commercial diet only and the probiotic-treated group (PROBIO) was fed twice per day with the commercial diet and twice per day with the lyophilized probiotic strain L. rhamnosus IMC 501, provided by Synbiotec (Camerino, Italy), via rearing water at a final concentration of 106 colony forming units/g (0.01 g/L), according to the manufacturer’s suggestions, for 28 days [19].

### 2.3. Behavioral Testing

*Zebrafish* shoaling behavior was evaluated for all 4 weeks of treatment in both control (CTRL) and probiotic treated (PROBIO) groups. Different behavioral scores were evaluated: (i) distance variance (DV), which was the shoal cohesion variance for each fish and represented the homogeneity of the distribution of fish within that shoal; (ii) occupied area (OA), which calculated the occupied area of all animals in a temporal unit on a two-dimensional plane, and (iii) water column position (CP), which represented the water column position (surface = 0) and indicated the deep preference of shoaling animals in the tank.

### 2.4. DNA Extraction and Bisulfite and Oxidative Bisulfite Conversion

Genomic DNA was extracted from tissue samples using the DNeasy Blood & Tissue Kit (QIAGEN, Hilden, Germany), following the manufacturer’s protocol. After quantification, 1000 ng of genomic DNA were converted with sodium bisulfite using an EZ DNA Methylation Kit (ZYMO RESEARCH, Irvine, CA, USA). In order to monitor the efficiency of the bisulfite conversion of DNA, the unmethylated control gene M13mp18, a synthetic gene with a known number of methylation sites, was converted, processed and sequenced together with the samples. Oxidative bisulfite conversion for 5-hmC detection was performed using the TrueMethyl oxBS module (Nugen, Tecan, CA, USA) following the manufacturer’s instructions. 

### 2.5. Amplicon Based Library Preparation 

Bisulfite-converted DNA was amplified in two steps of amplification. The first PCR was performed using FastStart High Fidelity PCR Systems (Roche) and the following primers were added to the primer sequences for the annealing of Nextera XT Index (Illumina) in the second step of PCR: *BDNF*—FW: 5′ gaatgtgaaTaaaaatgtTaaaag 3′; RV: 5′ taatAAactcccatAactAaA 3′ *Tph1A*—FW: 5′ atttgTtgtTaggaggaagattaag 3′; RV 5′ cacaacatcaaattctctacat 3′. We used some specific upstream adapters (FW: 5’ tcgtcggcagcgtcagatgtgtataagagacag 3’; RV: 5’ gtctcgtgggctcggagatgtgtataagagacag 3′). Both PCR steps were followed by the purification of amplicons with Agencourt AMPure XP beads (Beckman Coulter Genomics). Amplicons were quantified using Qubit^®^ 2.0 Fluorometer and the library was diluted to a final concentration of 8pM. Phix control libraries (Illumina) were combined with the normalized library (10% (*v*/*v*)) to increase the variability of base calling during sequencing. The amplicons’ library was subjected to sequencing using V2 reagent kits on the Illumina MiSeq system (Illumina). Paired-end sequencing was performed in 251 cycles per read (251 × 2). An average of approximately 100,000 reads/sample were obtained.

### 2.6. Sequence Handling and Bioinformatics Analyses

Paired-end sequences were first converted into FASTA format as previously described [28]. Sequences were then analyzed using the ad-hoc bioinformatic pipeline AmpliMethProfiler [26] (https://sourceforge.net/projects/amplimethprofile), specifically designed for deep-targeted bisulfite amplicon sequencing. As the output, the pipeline generates a summary file with information about the number of reads passing the filters, the methylation percentage of each C in the CpG sites, and the bisulfite efficiency for each C in non-CpG sites. ampliMethProfiler produces a tabular format file (BIOM format) containing the number of methylation profiles (epialleles) for all samples. The BIOM table was normalized for the same number of sequences/samples through a rarefaction procedure using QIIME, version 1.9.1.

### 2.7. Statistical Data Analysis 

Statistical tests were performed with QIIME, Excel and Graphpad Prism 7.0. Graphs were generated with Graphpad. Differences in average methylation were evaluated by 2-tailed unpaired parametric *t*-test in Graphpad assuming both populations have the same SD. Differences in single-CpG-site methylation were calculated by multiple *t*-test in Graphpad, followed by Holm–Sidak correction. In this study results were considered statistically significant when *p*-value < 0.05. A Pearson correlation test was used to assess the eventual relationship between DNA methylation data and behavioral scores.

## 3. Results and Discussion

### 3.1. Experimental System

**Figure 1:**
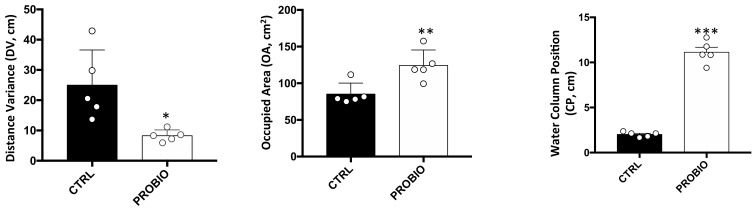
Behavioral parameters recorded in *Zebrafish* samples before and after probiotic treatment. Distance variance (DV), occupied area (OA) and water column position (CP) measurements in CTRL and RPOBIO groups. Statistical analysis has been performed using Student *t*-test (* *p* ≤ 0.05; ** *p* ≤ 0.01; *** *p* ≤ 0.001).

In order to investigate whether probiotic treatment may have an impact on DNA methylation in gut and brain cells, we took advantage of a *Zebrafish* experimental model previously characterized for gut microbiota, specific genes expressions and behavioral changes upon *L. Rhamnosus* administration. Some brain and gut tissues from the same experiment were stored at −80 °C, and are here utilized for methylation analyses. In brief, the feeding of the probiotic *L. rhamnosus* IMC 501 significantly altered the social and explorative behavior in these animals, as determined by several tests (Figure 1 and [19]). Moreover, among others, *BDNF* and *Tph1A* gene expression significantly changed in the gut and brain upon probiotic treatment (Supplementary Figure S1 and [19]). The gut microbial communities showed several changes in treated fishes. Particularly, at the phylum level, the Firmicutes strongly increased (4.10% CTRL, 28.75% PROBIO), while Proteobacteria decreased (17.24% CTRL, 9.01% PROBIO) in the treated group. The abundance of *Lactobacillus* (8.3%, about sixfold increase compared to untreated fishes) in the treated group indicated that *L. rhamnosus* colonized the gut. A significant increase in *Streptococcus* (about 25-fold increase in treated compared to untreated fish) was observed upon probiotic treatment, likely related to the symbiotic relationship between *L. Rhamnosus* and *S. thermophilus*. Additional information about fishes and treatment are reported in the Materials and Methods section. Full details on the experimental system used in this study are reported in Borrelli et al. [19]. The limitation of the present study is mainly represented by the limited number and amount of gut and brain tissues that could be retrieved by fishes belonging to the same, previously characterized, experimental system. For this reason, we selected a subset of five control and probiotic-treated fishes that showed behavioral changes. This subset was used to fully characterize the DNA methylation average, methylation levels at single-CpG sites and epiallele classes in the brain and gut at two genes (*BDNF* and *Tph1A*), and all DNA methylation analyses were correlated with specific behavioral scores.

Due to the limited amount of genomic DNA and bisulfite primer design difficulties, we chose to analyze the methylation state at two genes (*BDNF* and *Tph1A*) among those that showed treatment-related expression changes, as described in Borrelli et al. [19] 

### 3.2. Behavioural Changes Induced by Probiotic Administration in Zebrafish Samples

First, we recorded *Zebrafish* shoaling behavior for all 4 weeks of treatment in both the control (CTRL) and probiotic-treated (PROBIO) groups. Different behavioral scores were evaluated, as follows: (i) distance variance (DV), which was the shoal cohesion variance for each fish and represented the homogeneity of the distribution of fish within that shoal; (ii) occupied area (OA), which calculated the occupied area of all animals in a temporal unit on a two-dimensional plane, and (iii) water column position (CP), which represented the water column position (surface = 0) and indicated the deep preference of shoaling animals in the tank. We found that DV significantly decreased after probiotic treatment, while both OA and CP significantly increased in the PROBIO group. Thus, *L. Rhamnosus* administration strongly modified the shoaling behavior of *Zebrafish*. 

### 3.3. Tissue-Specific DNA Methylation at BDNF and Tph1A Promoter Regions in Zebrafish Gut and Brain

**Figure 2:**
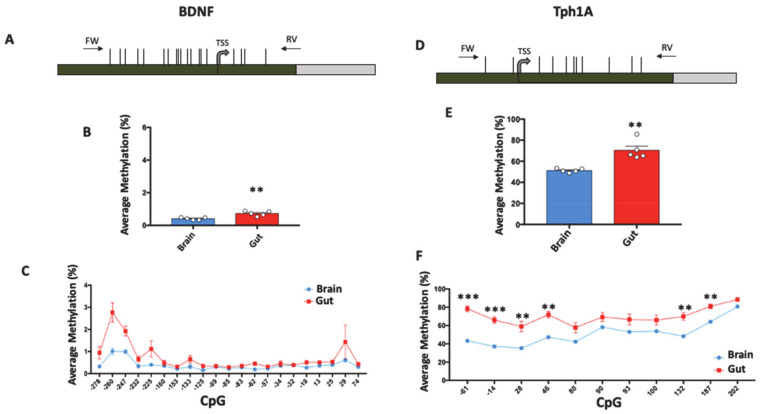
Tissue-specific DNA methylation of *BDNF* and *Tph1A* in gut and brain. (**A**) *BDNF* promoter region structure showing the position of analyzed CpGs (Horizontal black lines). The numbers of the CpG sites refer to the putative transcriptional start site (TSS), indicated with +1. Green box and gray box indicate promoter and the first intron, respectively. Positions of primers (FW: Forward; RV: Reverse) used for amplification procedure are reported. The *BDNF* sequence was retrieved by Ensembl with the following accession number: ENSDARG00000018817. (**B**) *BDNF* average methylation (%) in brain (Blue Box) and gut (Red Box) of untreated *Zebrafishes* is reported and indicated as the mean ± standard error of five samples. (**C**) Average methylation at single CpG site (%) at the *BDNF* promoter region in brain and gut is reported with blue and red lines, respectively, and indicated as the mean ± standard error of five samples. (**D**) *Tph1A* promoter region structure showing the position of the analyzed CpGs with numbers referring to the putative transcriptional start site (TSS). Green and gray boxes indicate promoter and the first intron, respectively. Black arrows at the top of the map specify the position of the primers used for bisulfite amplification. The *Tph1A* sequence was retrieved by Ensembl with the following accession number: ENSDARG00000029432. (**E**) Average methylation (%) at *Tph1A* in gut and brain of five control *Zebrafishes*. (**F**) Methylation level at single-CpG sites (%) in the *Tph1A* analyzed region. Comparison between brain and gut for both *Tph1A* and *BDNF* was performed using Student *t*-test (** p ≤ 0.01). Statistical analyses at single-CpG levels were performed using one-way ANOVA followed by Tukey’s multiple comparison post-hoc test (* *p* ≤ 0.05; ** *p* ≤ 0.01; *** *p* ≤0.001).

Then, we computed DNA methylation in the gut and brain tissues of five untreated *Zebrafish* at the promoter region of *BDNF* and *Tph1A* genes. For the *BDNF* gene, we analyzed a region of 417 bp, containing 21 CpG sites and encompassing the transcriptional start site (TSS) (Figure 2A). Despite this, the DNA methylation average was found to be very low both in the gut (0.72% ± 0.06, mean ± standard error) and in the brain (0.41% ± 0.034, mean ± standard error ). *BDNF* methylation levels were significantly (Student *t*-test; *p* = 0.003) higher in gut compared to brain (Figure 2B). We then investigated DNA methylation at the single-CpG level, in order to evaluate whether specific CpG sites differently contribute to the global methylation of the *BDNF* promoter (Figure 2C). We found that the methylation level at −260, −247, −125, −62 and −19 CpGs sites increased in gut compared to brain, but the results were not statistically significant. Most of the analyzed CpG sites presented very low methylation levels both in the gut and in the brain (not exceeding 1% of methylation): −260 and −247 CpGs carried the highest values of DNA methylation in both analyzed tissues.

We then performed methylation analyses on the *Tph1A* promoter in the brain and gut of the same five control *Zebrafish*. We analyzed a region of 318 bp, containing 11 CpG sites and encompassing the TSS (Figure 2D). We found that the average DNA methylation of the *Tph1A* gene was significantly (Student *t*-test; *p* = 0.0016) higher in the gut (70.38% ± 4) compared to the brain (51.24% ± 0.8) (Figure 2E), and the higher levels of methylation in the gut were conserved in all analyzed CpG sites (Figure 2F). Specifically, all CpG sites, with the exception of +80, +90, +93 and +100 CpGs, showed a significantly (Multiple *t*-test followed by Holm–Sidak correction; *p* < 0.01) higher degree of methylation in gut compared to brain. 

In order to evaluate whether differences in DNA methylation between the gut and brain of control *Zebrafish* were associated with different expressions of *BDNF* and *Tph1A* genes, we utilized and re-analyzed previously published mRNA expression data [19] of *BDNF* and *Tph1A* (Supplementary Figure S1) from the same *Zebrafish* samples used for the here-presented methylation analyses. The mRNA expression analysis showed that both *BDNF* and *Tph1A* were more expressed in brain compared to gut, correlating with the higher promoter methylation degree of both genes in the gut. Therefore, DNA methylation at the *BDNF* and *Tph1A* genes regulates the expression levels of these genes in a specific spatial manner. Because the methylation levels at *BDNF* were very low, it is likely that mechanisms other than DNA methylation are predominant in *BDNF* regulation and are the main cause of differential expression between the brain and the gut. On the contrary, it is likely that for *Tph1A*, DNA methylation plays a critical role. Up to now, to our knowledge, no studies have addressed the tissue-specific epigenetic control carried out by the DNA methylation of brain-related genes in *Zebrafish*. 

### 3.4. Ultra-Deep DNA Methylation Analyses at BDNF Promoter Region upon Probiotic Administration in Gut and Brain Tissues

Since treatment with *L. rhamnosus* was previously shown to increase *BDNF* mRNA expression [19] in the gut and brain of *Zebrafish* models, we analyzed *BDNF* promoter methylation in both tissues of treated samples (PROBIO), compared with the methylation state observed in untreated *Zebrafish* samples (CTRL). 

**Figure 3:**
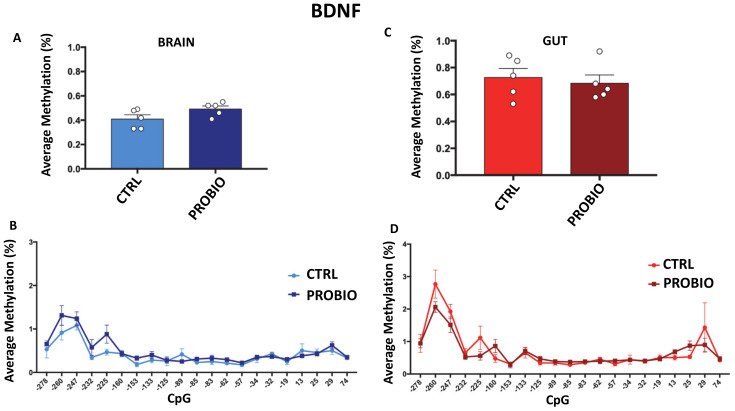
DNA methylation of *BDNF* in probiotic-treated and untreated *Zebrafishes* in the gut and brain. (**A**) Average methylation (%) of *BDNF* in the brain of untreated (CTRL) and probiotic-treated (PROBIO) *Zebrafishes*. Statistical analysis was performed using Student *t*-test. (**B**) Methylation level (%) at single-CpG sites was shown for the brain of CTRL and PROBIO groups. (**C**) *BDNF* average methylation (%) in the gut of untreated (CTRL) and probiotic-treated (PROBIO) *Zebrafishes*. Statistical analysis was performed using Student *t*-test. (**D**) Single-CpG methylation (%) at the *BDNF* promoter in the gut of CTRL and PROBIO groups. Statistical analyses were performed using one-way ANOVA followed by Tukey’s multiple comparison post-hoc test.

As shown in Figure 3A,C, the *BDNF* analyzed region still exhibited very low levels of methylation in the probiotic-treated group (ranging from 0.4% to 0.8% of average methylation). No significant differences were found between CTRL and PROBIO groups both in brain and in gut tissues. No significant methylation differences at the single-CpG level were found between the CTRL and PROBIO groups (Figure 3B,D).

According to our data, the previously observed [19] increase in *BDNF* expression in the brain as well as the decreased expression in the gut following probiotic treatment seem not to depend on the methylation state of the *BDNF* promoter. This effect may be expected since the *BDNF* promoter is characterized by a dense CpG island that, as is widely described [29,30], is generally poorly methylated.

### 3.5. Tph1A Promoter Methylation and 5′-Hydroxymethylation in Zebrafish Gut and Brain upon Probiotic Treatment

**Figure 4:**
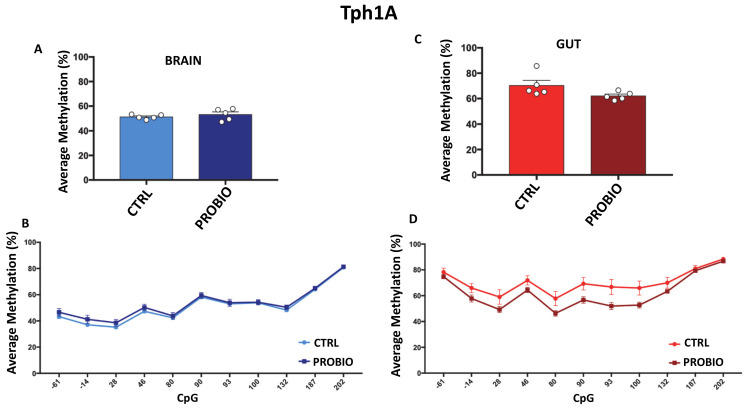
Comparison of *Tph1A* DNA methylation between CTRL and PROBIO groups in gut and brain. (**A**) Average methylation (%) of *Tph1A* in the brain of untreated (CTRL) and probiotic-treated (PROBIO) *Zebrafishes*. Statistical analysis was performed using Student *t*-test. (**B**) Methylation level (%) at *Tph1A* single-CpG sites was shown for the brain of CTRL and PROBIO groups. (**C**) *Tph1A* average methylation (%) in the gut of untreated (CTRL) and probiotic-treated (PROBIO) *Zebrafishes*. Statistical analysis was performed using Student *t*-test. (**D**) Single-CpG methylation (%) at the *Tph1A* promoter in the gut of the CTRL and PROBIO groups. Statistical analyses were performed using one-way ANOVA followed by Tukey’s multiple comparison post-hoc test.

We then analyzed DNA methylation levels at the promoter region of the *Tph1A* gene (Figure 2D) in the CTRL and PROBIO groups in gut and brain tissues. We found no significant differences in average methylation between CTRL and PROBIO in brain tissues (Figure 4A), although a slight increase in methylation level in the PROBIO group was found (CTRL = 51.2% ± 0.8; PROBIO = 53.2% ± 2.1, mean ± standard error). Conversely, a 10% decrease in average methylation between the CTRL group (70.38% ± 4, mean ± standard error) and the PROBIO group (62.1% ± 1.4, mean ± standard error) (Figure 4C) was observed in the gut, although this difference was not significant (Student *t*-test; *p* = 0.08), probably due to the low number of samples (*n* = 5). The decrease in DNA methylation in the gut of the PROBIO group was observed in all analyzed CpG sites (Figure 4D), but also in this case, there were no significant differences (Multiple *t*-test followed by Holm–Sidak correction). These results indicate that the changes in *Tph1A* and *BDNF* mRNA expression observed in the gut and brain upon probiotic administration [19] may be related to several other regulatory mechanisms, such as chromatin remodeling or miRNA. We next asked whether the presence of 5-hydoxymethylcytosine (5-hmC) on the promoter region of *Tph1A* in the probiotic-treated *Zebrafishes* is associated with an increase in the mRNA expression of the gene in the gut and the brain as a result of probiotic treatment. Since bisulfite analysis could not distinguish between 5-methylcytosine (5m-C) and 5-hmC, we performed oxidative bisulfite sequencing at *Tph1A* in CTRL and PROBIO gut and brain tissues. We found a very low presence of 5-hmC with no differences between the two groups and the two areas (data not shown).

### 3.6. Epiallele Classes Analyses of Tph1A Promoter Region in Gut and Brain of Treated and Untreated Zebrafish Samples

Since brain and gut tissues are a heterogeneous complex of cells having different functions, the perturbation of the gut microbiota through administration of *L. rhamnosus* may affect DNA methylation at only a subpopulation of probiotic-responsive cells, rather than exerting a general effect on brain or gut cells.

Therefore, we performed a high-resolution methylation analysis that allowed us to highlight cell-to-cell methylation differences at a single-molecule level. Indeed, this approach mimics a single-cell analysis since it provides “epiallele” distribution profiles. As we and others have previously reported in detail [28,31,32,33], the term “epiallele” refers to a specific arrangement of methyl-CpG in a given gene region. Combining together all the epialleles that bear the same number of methylated CpGs regardless of their position, it is possible to obtain the amount of a specific “epiallele class”. 

**Figure 5:**
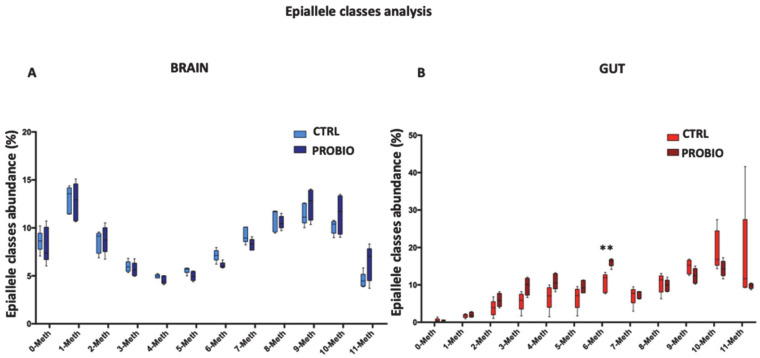
Epiallele classes distribution at the *Tph1A* promoter region in the gut and brain of CTRL and PROBIO groups. Boxplots show the relative abundance of different epiallelic classes (from 0 to 11 methyl-cytosine per molecule) in CTRL and PROBIO group and in (**A**) brain and (**B**) gut. Statistical analysis was performed using Multiple *t*-tests without correction. (** *p* ≤ 0.01).

We here applied “epiallele classes” distribution analyses, in order to evaluate whether specific epiallele classes at the *Tph1A* gene may be enriched or depleted in treated compared to untreated *Zebrafish* samples. We excluded from our analyses the *BDNF* promoter region, since the very low methylation level of the gene does not allow this kind of analysis. First, we performed epiallele classes analysis on the *Tph1A* promoter region, comparing CTRL and PROBIO groups in the brain and gut (Figure 5). A clear reshuffling of methylation classes appeared in the brain after probiotic administration (Figure 5A). The frequency of 0-, 1-, 2- and 3-Meth classes was found to be very similar in the CTRL and PROBIO groups. A depletion of 4-, 5-, 6- and 7-Meth classes was detected in the PROBIO group compared to the CTRL group. The converse held for the 9-, 10- and 11-Meth classes (higher in PROBIO group). Thus, even though the global average methylation of CTRL and PROBIO groups showed no differences in the brain, after probiotic administration a reconfiguration of specific methylation classes occurred. In the gut, the decrease in methylation found in the PROBIO group was due mainly to a significant (Multiple *t*-test, *p* = 0.002) increase in the 6-Meth class as well as a slight, non-significant increase in the 3-, 4-, and 5-Meth classes and a concomitant decrease in 10- and 11-Meth classes (Figure 5B). 

As such, the *Tph1A* promoter undergoes a remodeling of methylation trajectories upon probiotic administration, and the major shifts happen in the intermediate classes of epialleles at the *Tph1A* promoter. Although we cannot associate this methylation remodeling with the observed changes in the mRNA expression of the *Tph1A* gene after probiotic administration, we speculate that the perturbation of gut microbiota drives methylation/demethylation events in a specific and selective manner. These data demonstrated that the administration of *Lactobacillus rhamnosus* changed the methylation structure and profile of the *Tph1A* promoter, and allowed us to distinguish the two analyzed groups both in the brain and gut. 

### 3.7. Correlation between Tph1A Methylation Data and Behavioral Changes in Treated and Untreated Zebrafish

**Figure 6:**
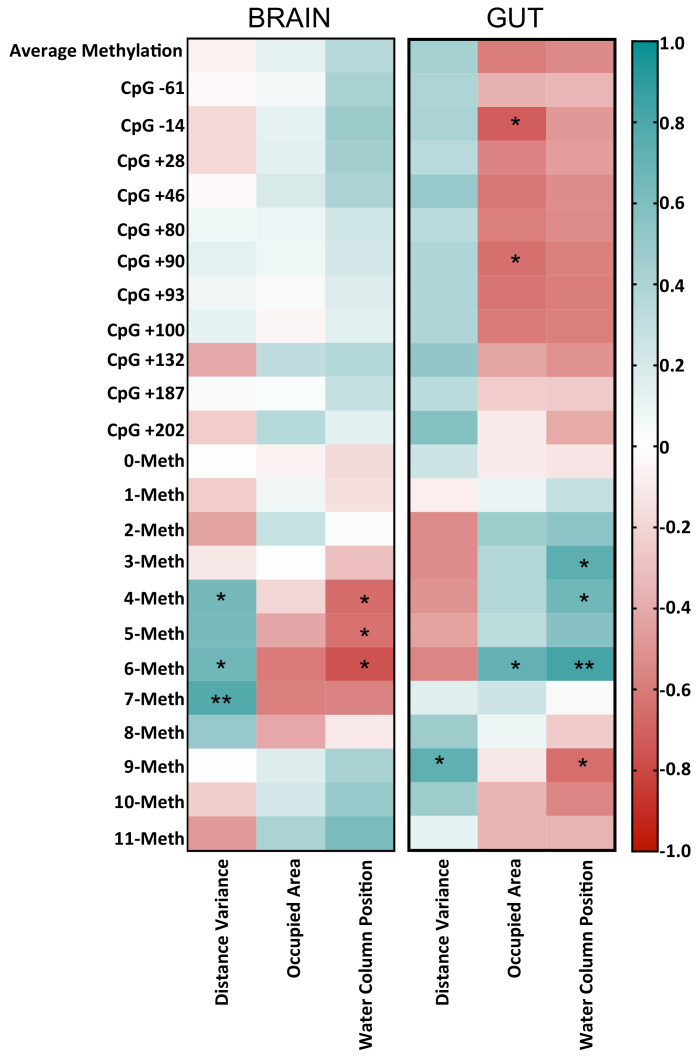
Correlogram showing Pearson correlation between DNA methylation data and behavioral parameters. The graph reports r values derived from Pearson correlation between average methylation, single-CpG methylation, epiallele classes and distance variance, occupied area and water column positions. The scale color from blue to red indicates a positive to negative correlation (−1 ≤ r ≥ 1). Statistical analyses were performed using a Pearson correlation test (* *p* ≤ 0.05; ** *p* ≤ 0.01).

It has been demonstrated in Zebrafish that the major effects observed upon probiotic treatments are related to the behavioral aspects, the development, and the physiology of reproductive system in the zebrafish model, acting on insulin-like growth factors-I (igfI) and -II (igfII), peroxisome proliferator activated receptors-α and -β, (pparα,β) vitamin D receptor-α (vdrα) and retinoic acid receptor-γ (rarγ) [34]. We and others [2,6,7,9,19,35] previously demonstrated that the administration of probiotics to Zebrafish ameliorated their explorative behavior and increased the mRNA levels of specific genes involved in brain functioning. However, to our knowledge, no studies correlated modifications in DNA methylation with behavioral changes after microbiota modulation. Based on the fact that L.Rhamnosus administration showed an alteration in behavior (Figure 1), and microbiota modulation likely had an effect on the DNA methylation of Tph1A gene, especially in epiallele class distribution (Figure 5), we decided to correlate the behavioral scores for the CTRL and PROBIO groups (Figure 1) with the DNA methylation average, the single-CpG methylation and the epiallele classes at the *Tph1A* gene in the brain and gut of treated and untreated *Zebrafish*. We reported all results from the Pearson correlation in the Supplementary Table S1. As shown in the heatmap (Figure 6), several positive and negative significant correlations were found in both tissues. In the brain, intermediate epiallele classes, such as 4-, 6- and 7-Meth, positively and significantly correlated with DV. These specific behavioral scores significantly decrease after probiotic treatment (Figure 1). A non-significative reduction in 4-, 6- and 7-Meth epiallele classes was also found in the brain of fishes upon probiotic administration (Figure 6). The presence of a significant positive correlation between these specific epiallele classes and the distance variance score let us speculate that the decrease in 4-, 6- and 7-Meth epiallele classes in the PROBIO group is strictly associated with the probiotic administration. Moreover, the 4-, 5- and 6-Meth classes showed a significant negative correlation with CP score. Additionally, in this case, the decrease in these specific epiallele classes after probiotic treatment was well associated with a significant increase in the CP score in the brain of treated fishes (Figure 1 and Figure 6).

In the gut, some significant correlation between behavior and methylation emerged also at single-CpG levels, as well as in epiallele classes. We found a significant negative correlation between OA and methylation at CpG −14 and CpG +90. Additionally, CP and 9-Meth class also negatively and significantly correlated. Interestingly, methylation at CpG −14 and CpG +90 slightly decreased in the PROBIO group, while OA significantly increased after treatment. Accordingly, the 9-Meth epiallele class decreased in the PROBIO group (Figure 6), while CP increased upon probiotic administration (Figure 1). We also identified a significant positive correlation between 3-, 4- and 6-Meth and CP scores, all values that increased after *L. rhamnosus* treatment (Figure 1 and Figure 6). Moreover, significant positive associations between the 6-Meth epiallele class and OA, and between 9-Meth and DV, were found, connected to an increase in both factors upon probiotic administration. The correlation analysis carried out here highlights the existence of a probiotic-induced effect on DNA methylation at the *Tph1A* promoter. As shown in Figure 1, the behavioral scores measured for the *Zebrafish* samples used in the present paper drastically and significantly changed after probiotic administration. Conversely, DNA methylation at the *Tph1A* promoter showed no significant changes in treated fishes. However, the presence of a significant correlation between behavioral changes and DNA methylation variation indirectly suggests that the probiotic has an effect on the methylation of *Tph1A* in the brain and in the gut.

## 4. Conclusions

In the present study, we showed that the administration of *L.rhamnosus* leads to the remodeling of the DNA methylation code at the *BDNF* and *Tph1A* promoter genes in the gut and brain of *Zebrafishes*. This methylation re-shuffling correlated with changes in mRNA expression in both gut and brain tissues, demonstrating that changes in microbiota composition may affect the host epigenetic landscape leading to long-lasting effects on specific gene regions. 

A full understanding of these mechanisms is crucial to developing a microbiota-based therapeutic strategy for psychiatric diseases, and for the discovery of alternative pathways and substrates to treat brain disorders that do not respond to available drugs. In this context, the *Zebrafish* model has great potential applicability in studying microbiota–gut–brain interactions and in a system to screen several prebiotics, probiotics and post-biotic metabolites. In order to decrypt the extent to which environmental effects, especially gut microbiota perturbation, can provoke epigenetic responses, it will be necessary in future to extend this investigation to other relevant genes potentially involved in the microbiota–gut–brain axis. Moreover, since probiotic effects likely occur only in subpopulations of cells, causing the observed changes in expression levels, single-molecule analysis, being a proxy of single cell analysis, has turned out to be a promising tool for future investigations. Nevertheless, using these tools, we were able for the first time to observe and describe fine changes in methylation profiles, derived from probiotic treatment, that significantly correlated with behavioral changes induced by probiotic administration. For these reasons, we are confident that further investigations will provide new insights into the complex topic of epigenetics’ role in the microbiota–gut–brain axis. 

## Data Availability

All raw data have been deposited in a public database (ENA) under accession number: PRJEB42598 and no restrictions will be applied.

## Supplementary Materials

The following are available online at https://www.mdpi.com/2218-273X/11/2/142/s1, Figure S1: mRNA expression levels of BDNF and Tph1A genes in brain and gut of untreated Zebrafish, Table S1: R values derived from correlation between behaviour and methylation at Tph1A gene.
