# Supplementary material for: DNA Methylation Profiles of Tph1A and BDNF in Gut and Brain of L. Rhamnosus-Treated Zebrafish
Source: Biomolecules. 2021 Jan 22;11(2):142. doi: 10.3390/biom11020142 (PMC7911505; doi:10.3390/biom11020142)

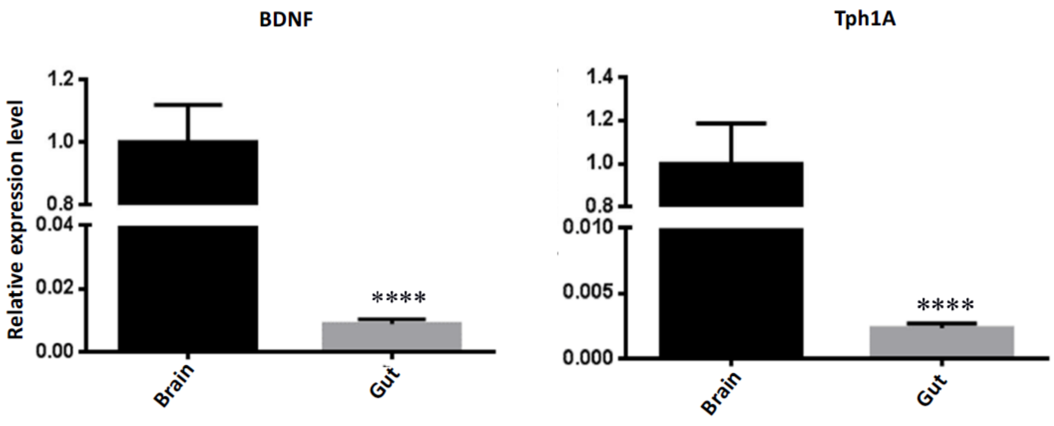

Figure 1. mRNA expression levels of BDNF and Tph1A genes in brain and gut of untreated Zebrafish.

Table 1. R values derived from correlation between behaviour and methylation at Tph1A gene.

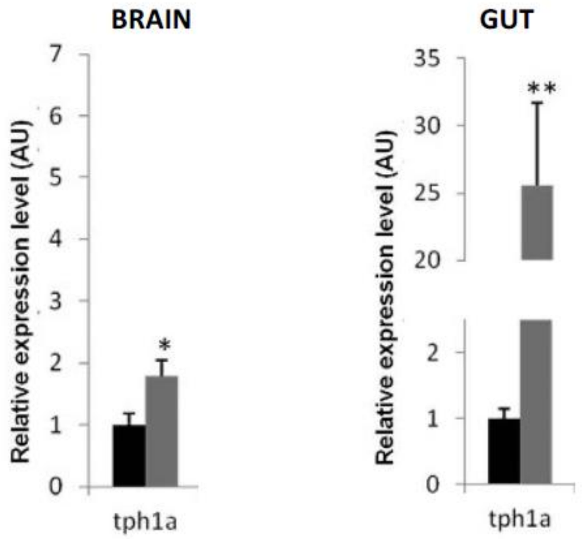

Supplement: Supplementary file 1 [file biomolecules-11-00142-s001.pdf]
